# SM2-PRE+: A Lightweight Pairing-Free Proxy Re-Encryption Scheme for Secure IoMT Healthcare Data Sharing

**DOI:** 10.3390/s26185761

**Published:** 2026-09-10

**Authors:** Shuanggen Liu, Mingxing Zhu, Xu-An Wang, Ziqi Fan, Xinyu Zhou

**Affiliations:** 1 School of Cyberspace Security, Xi’an University of Posts and Telecommunications, Xi’an 710121, China; liushuanggen201@xupt.edu.cn (S.L.); z1770850505@163.com (M.Z.); fine@stu.xupt.edu.cn (Z.F.);; 2 Key Laboratory of Network and Information Security, Engineering University of People’s Armed Police, Xi’an 710086, China

**Keywords:** Internet of Medical Things, sensor data security, healthcare data sharing, proxy re-encryption, SM2 algorithm

## Abstract

With the rapid development of the Internet of Medical Things (IoMT), wearable sensors and intelligent medical terminals continue to generate a large amount of sensitive medical data, which needs to be uploaded to the cloud platform for storage and sharing. However, IoMT devices usually have the characteristics of limited computing resources and limited energy, and it is difficult for traditional high-complexity encryption schemes to meet the requirements of security and efficiency. In addition, the existing Proxy Re-Encryption (PRE) scheme has the risk of authorization transfer, and it is difficult to achieve fine-grained and secure sharing of medical data. In response to the above problems, this paper proposes a lightweight non-pairing proxy re-encryption enhancement scheme SM2-PRE+ based on the SM2 algorithm, which is used for the secure sharing of IoMT medical sensing data. The scheme combines the SM2 elliptic curve cipher algorithm and the SM4 symmetric encryption algorithm to achieve data protection through a double-layer key structure, and it uses the re-encryption mechanism of message binding to enhance the authorization control ability. Compared with the traditional PRE scheme, the proposed scheme avoids bilinear pairing operations, reduces the computing overhead of resource-limited equipment, and supports collusion-resistant and authorization non-transferability. Security analysis shows that the scheme meets the security requirements of IND-CCA under the Random Oracle Model (ROM). Performance analysis results show that SM2-PRE+ has low computing overhead and storage burden, which is suitable for wearable medical devices, intelligent sensing terminals, and cloud-assisted IoMT data sharing scenarios.

## 1. Introduction

The cloud-assisted medical platform combines cloud computing, the Internet of Medical Things (IoMT), and medical data management technology [1,2]. It can realize centralized aggregation of medical data, cross-terminal sharing, and intelligent analysis. It also assists clinical decision-making. In the overall architecture design, the system adopts a distributed storage method. It collects various medical data from hospitals, specialized clinics, and community health centers at all levels, including different types of files such as electronic medical records, imaging data, and laboratory results. Meanwhile, with the development of IoMT, wearable sensors and intelligent medical terminals have become important sources of healthcare data [3]. They continuously generate physiological information such as heart rate, blood pressure, and other personal health records.

These scattered data are then sorted out, standardized, and stored in a structured manner. By using the supported data transmission platform, data between different departments and medical institutions can be immediately interoperable [4]. For ordinary patients, they only need to encrypt their mobile phones and upload personal medical data to the cloud. Then they can view their health records anytime and anywhere, without being affected by the lack of storage space on local devices. Practitioners who have passed identity verification can obtain the required patient data through secure channels [5]. This allows them to smoothly carry out medical work such as remote consultation and precise treatment.

However, we also know that there are still many technical challenges and management vulnerabilities in the process of sharing encrypted medical data in the cloud [2]. Everyone has strict requirements for their own access to medical records. Most people only allow their attending doctor to view their information. They never allow doctors to privately transfer this access to interns, personnel of other departments, or external institutions, because this is tantamount to leaking the patient’s personal information. Therefore, we have put forward the core requirement of non-transferable authority [6,7]—that is, the permission of the doctor authorized by the patient cannot be transferred to others. In addition, IoMT-enabled healthcare systems introduce additional security challenges due to the large number of distributed sensing devices and resource-constrained terminals. Cloud-based medical systems need to deal with various network security risks. On the one hand, it is necessary to prevent semi-trusted cloud agents from stealing or leaking private data during transmission. On the other hand, it is also necessary to prevent low-authorized users from colluding with each other to bypass the system verification mechanism and illegally obtain data. Whether it is the collusion of internal service providers with external hackers or the joint breakthrough of permission verification by multiple malicious users, these real attack risks put very high demands on the privacy protection capabilities of cloud-assisted IoMT healthcare system systems.

Proxy re-encryption (PRE) is currently the mainstream encryption technology for data authorization and secure sharing [8]. It allows the proxy server to complete ciphertext conversion without reading the original plaintext, so that the designated user can decrypt and use the data normally. However, when applied to the cloud-assisted IoMT healthcare system scenario, its shortcomings are obvious. First, the security of the re-encryption key is insufficient, making it easy for proxy and receiving users to collude and abuse it, which leads to arbitrary circulation of data permissions. Second, medical data are large in volume and frequently updated. Traditional schemes need to generate a separate key for each piece of data, which greatly increases computing and storage pressure—a problem that becomes more challenging in IoMT environments where sensing devices usually have limited computational capability and energy resources. Finally, traditional PRE cannot accurately authorize individual data, making it difficult to meet flexible and differentiated access needs in clinical diagnosis and treatment. For this reason, the academic community has proposed an optimized PRE+ mechanism [9]. It changes the key generation logic by transferring the key generation right from the data client to the encryptor, and fundamentally realizes non-transferable permissions while supporting refined data control. However, existing PRE+ schemes still have obvious shortcomings. They fail to properly address security risks introduced by key reuse. Moreover, they cannot fully satisfy special regulatory rules and business requirements from the medical industry. Therefore, they are difficult to deploy directly for real-world cloud-assisted IoMT healthcare data-sharing scenarios [10]. Based on these observations, this study explores how to combine the advantages of the SM2 encryption algorithm with the PRE+ framework for IoMT-based healthcare data sharing. The motivation comes from a practical need—that sensor-generated medical data should be securely shared among authorized healthcare entities without sacrificing the owner’s control over authorization. Therefore, we have designed an unpaired proxy re-encryption enhancement scheme based on SM2, called SM2-PRE+.

### 1.1. Related Work

With the rapid development of cloud computing and intelligent medical services, a large amount of sensitive medical information, including electronic health records (EHRs), medical images, and physiological monitoring data, is increasingly stored and shared through the cloud platform. Although cloud-based medical systems provide scalable storage and convenient data access, outsourcing sensitive medical information to semi-trusted cloud servers brings significant privacy and security challenges. Therefore, the design of an efficient encryption mechanism that can achieve secure data sharing without disclosing plaintext information has become an important research issue. Proxy re-encryption (PRE) allows a semi-trusted agent to convert ciphertext from one user to another without accessing the underlying plaintext, which has been widely studied as an effective encryption original language for secure cloud data sharing. The concept of PRE was originally proposed by Blaze and others [8], which allowed the agent to convert a ciphertext encrypted with one key into a ciphertext that could be decrypted with another key. The pioneering work has laid the basic framework for encrypted data entrustment. Subsequently, Athense et al. [6] improved the practicality of PRE and showed its application in secure distributed storage systems. In order to enhance privacy protection in the process of ciphertext conversion, Ateniese et al. [11] introduced the key privacy attribute to prevent unauthorized entities from inferring the identity of the recipient from the re-encrypted ciphertext.

Canetti and Hohenberger [12] established the CCA security model of PRE, while Boneh et al. [13] further strengthened the security of selected ciphertexts through an identity-based encryption framework. Zengin et al. [14] studied the one-way PRE of selected ciphertext security based on asymmetric pairing and achieved a strong confidentiality guarantee. With the increasing demand for scalable identity management, identity-based password technology has been integrated into the PRE system. Boneh [15] proposed a practical identity-based encryption setup, which provides a theoretical basis for identity-oriented encryption applications. Based on this concept, Green and Ateniese [16] proposed identity-based proxy re-encryption (IB-PRE), eliminating the need for traditional public key certificates and simplifying key management in distributed environments. However, many old PRE and IB-PRE schemes rely on bilinear pairing operations, which leads to high computing costs and limits their deployment in medical and Internet of Things environments with limited resources. In order to enhance the security of advanced attacks, Libert and Vergnaud [17] have researched a PRE scheme that can resist adaptive ciphertext selection and formulated a strict security definition for actual deployment. Shao et al. [18] proposed a general one-way PRE technology, which can convert ciphertext multiple times while maintaining security.

With the increasing popularity of cloud-based medical systems, PRE has been widely studied to protect the sharing of medical data between patients, medical service providers, and research institutions. Unlike traditional encryption methods that require direct exchange of keys, PRE allows data owners to entrust access to encrypted data without disclosing plaintext information, which makes it particularly suitable for multi-party health scenarios. Duan et al. [1] proposed a proxy re-encryption scheme based on attributes and multi-permission institutions for collaborative sharing of electronic health files in the Web 3.0 environment. By introducing multiple permission institutions and attribute-based access policies, the program achieves fine-grained authorization among different participants in the medical industry. However, attribute-based mechanisms often require complex policy evaluation and additional encryption operations, which may increase the computational burden of lightweight medical equipment. Li et al. [3] designed a proxy re-encryption scheme with equivalence test for the Internet of Things medical systems, which can effectively compare encrypted data while protecting privacy. Liu et al. [19] also proposed a document-based PRE+ scheme to securely share medical information, which solves the challenges of authentication and key management in the medical environment. Blockchain technology is also used in combination with electronic health records to improve the transparency, integrity, and verifiability of health data exchange. Chen et al. [5] proposed a blockchain-based electronic medical record sharing framework, which uses proxy re-encryption, in which the blockchain provides anti-tampering audit, while PRE allows the security entrustment of encrypted data. Similarly, Liu et al. [20] has studied the blockchain-based medical data sharing mechanism to improve access control and data integrity. Pei et al. [4] have studied the application of blockchain-based PRE mechanism in IoMT data sharing in depth, demonstrating the potential of PRE in protecting sensitive medical information generated by distributed medical devices.Blockchain-assisted proxy re-encryption has also been explored in vehicular communication systems [21], and a CCA-secure pairing-free broadcast proxy re-encryption scheme has been proposed for VANETs [22], enabling secure sharing of encrypted information among dynamic IoT entities.

The medical big data environment also needs a flexible and scalable privacy protection mechanism. Existing research on the security management of medical big data shows that the cryptographic entrustment mechanism can improve the level of privacy protection in large-scale medical information systems. Nevertheless, it is still a huge challenge to strike a balance between security functions and computing efficiency. In the actual cloud-assisted IoMT healthcare system, different users usually need different access rights, depending on their roles, responsibilities, and relationship with patients. Therefore, traditional PRE solutions with simple authorization functions are not enough to handle complex authorization needs. In order to solve this problem, researchers introduced a conditional, attribute-based, and revocable PRE mechanism. Chu and Tzeng [23] constructed an identity-based proxy re-encryption scheme without random oracles, simplifying key management in distributed environments. By embedding authorization requirements into the re-encryption process, conditional PRE provides more flexible access control than traditional PRE. Weng et al. [24] further studied the conditional PRE under the assumption of stronger security and improved the security based on the conditional ciphertext delegation. Pareek et al. [7] studied a very fine access control mechanism based on PRE, so that data owners can formulate detailed access policies for encrypted data sharing. The efficient conditional PRE scheme [25] also tries to reduce computing overhead while maintaining flexible authorization capabilities. Dynamic user management is another important need in cloud-assisted IoMT healthcare system environments where medical personnel, researchers, and service providers may frequently join or leave the system. Ge et al. [26] proposed an revocable proxy broadcast re-encryption mechanism, which allows the sharing of encrypted information between multiple recipients and supports user revocation. Liang et al. [27] has conducted an in-depth study of identity-based revocable agent re-encryption, so that users can be effectively removed without disclosing the content of the ciphertext. However, how to update the key effectively and minimize the communication burden is still a challenge.

Even if PRE provides secure delegation functions, many existing schemes still have a large amount of calculations because of the use of bilinear pairs and complex cryptographic primitives. This limitation is particularly problematic in the healthcare Internet of Things environment, as wearable devices, sensors, and edge terminals usually have limited computing power and energy resources. Therefore, those lightweight PRE schemes that reduce the computing burden while maintaining security are attracting more and more attention. Yan et al. [10] proposed a pairing-free certificate-based proxy re-encryption plus scheme. By eliminating expensive bilinear pairing operations, it allows efficient delegation of ciphertexts. Their research shows that elliptical curve cryptography technology can be used to achieve lightweight PRE. Sun et al. [28] proposed a lightweight PRE scheme for industrial Internet of Things systems, with the aim of reducing encrypted computing while maintaining the security of data transmission between devices. Li et al. [29] discussed the cloud-assisted Internet of Things data sharing framework using PRE, indicating that encrypted information can be safely delegated between heterogeneous Internet of Things devices and cloud servers. In addition, the blockchain-based Internet of Things privacy protection mechanism [30,31] studies the combination of distributed ledger technology with encryption entrustment mechanism to improve the integrity and responsibility of data. However, the blockchain-based approach may increase additional storage and communication overhead. The comprehensive investigation and analysis of the PRE scheme [2] also points out that there is always a trade-off between security, functionality, and efficiency, which indicates that the future PRE design should achieve lightweight computing, flexible authorization, and practical deployment capabilities at the same time.

In addition to efficiency optimization, recent research also focuses on improving the security functions of the PRE scheme, including post-quantum security, verifiability, fairness, and advanced ciphertext conversion capabilities. Kirshanova [32] studies the lattice-based PRE constructs, which are based on the lattice difficulty hypothesis and provide potential post-quantum security. Dutta et al. [33] proposed a grid-based identity preprocessing structure, which can resist homogeneous attacks and provide stronger protection against proxy user collusion attacks. Singh et al. [34] explored the grid-based PRE+ mechanism, while Fan et al. [35] studied the grid-based proxy re-encryption and re-signature construction. Although grid-based schemes seem promising in resisting quantum attacks, they often have some problems, such as large encrypted messages, complex parameter settings, and high computing costs. In order to improve trust in a semi-trusted cloud environment, Tang et al. [36] proposed a verifiable PRE scheme based on attributes, so that users can verify whether the agent has correctly executed the ciphertext conversion. Ge et al. [37] further studied the fair and verifiable PRE mechanism to achieve accountability and prevent malicious proxy behavior. Wang et al. [38] studied the blockchain-based privacy protection mechanism, combined with encryption proxy technology, and demonstrated the potential of integrating PRE into the decentralized infrastructure.Wang et al. [39] proposed IBPRE+ for message-level fine-grained proxy re-encryption and weak non-transferable social cloud authorization, overcoming drawbacks of traditional IB-PRE. In addition, the conditional PRE scheme for the federal learning environment [40] shows the potential of ERP in collaborative computing scenarios that protect privacy.

In recent years, PRE+ has emerged as an enhanced paradigm. It aims to overcome the limitations of the traditional PRE delegation model. Wang et al. [9] proposed a PRE+ framework, which improves the flexibility of authorization and reduces the risk of traditional re-encryption key delegation. Zhou et al. [41] studied an unpaired PRE scheme for the secure sharing of medical data, demonstrating the feasibility of lightweight deployment of PRE in health scenarios. Despite significant progress, existing PRE and PRE+ programs still face some challenges in the actual cloud-assisted IoMT healthcare system data sharing environment. First of all, many existing solutions rely on bilinear pairing, attribute-based processing, or network-based password primary, which will bring a high computing and storage burden. These designs are difficult to deploy in a medical Internet of Things environment with limited resources that include wearable devices and edge terminals. Secondly, the traditional PRE scheme usually generates re-encrypted keys based on the long-term secret information of the data owner, which makes it difficult to prevent unauthorized transfer or secondary delegation of access. Third, existing healthcare-oriented PRE schemes mainly focus on confidentiality and access control, while insufficient attention has been given to message-level authorization, collusion resistance, and non-transferability. To address these challenges, this paper proposes an SM2-based Proxy Re-Encryption Plus (SM2-PRE+) scheme for secure cloud-assisted IoMT healthcare system data sharing. Different from conventional PRE mechanisms, the proposed scheme introduces a lightweight PRE+ framework where re-encryption authorization is generated based on specific sharing relationships rather than relying solely on traditional delegation models. By leveraging the SM2 elliptic curve cryptographic algorithm and efficient symmetric encryption techniques, the proposed scheme eliminates expensive pairing operations and reduces computational overhead. Furthermore, the proposed design provides message-level authorization, collusion resistance, and non-transferability, enabling secure and efficient cloud-assisted IoMT healthcare system data sharing in practical IoMT environments.

### 1.2. Motivation

Cloud-based healthcare information systems with IoMT devices feature both high security requirements and high-frequency data sharing. With the increasing deployment of wearable sensors and intelligent medical terminals, more sensor-generated healthcare data need to be securely stored and shared through cloud platforms. They not only require strict protection of medical data privacy but also need to ensure the efficiency and scalability of cross-institutional data interaction. Moreover, medical data management poses challenges to the scheme in terms of security, non-transferability, and anti-collusion attack capabilities.

Most existing proxy re-encryption schemes only achieve weak Chosen Plaintext Attack (CPA) security. For cloud-assisted IoMT healthcare scenarios, it is critical to prevent collusion between proxies and malicious recipients aimed at stealing the sender’s private key. Meanwhile, traditional proxy re-encryption schemes struggle to satisfy strict regulatory requirements for medical systems. Meanwhile, the resource constraints of IoMT devices further increase the difficulty of designing efficient and lightweight security schemes. Existing proxy re-encryption schemes cannot effectively solve the problems in cloud-based healthcare information systems.

The SM2 algorithm has significant advantages in security and efficiency, and the nontransferability and fine-grained control features of the PRE+ scheme are highly compatible with the security needs of medical data sharing. The combination of SM2 and PRE+ can provide an effective solution for secure sharing of IoMT-generated healthcare data. The integration of the two can provide a solution that balances security and sharing for cloud-based healthcare systems. Therefore, designing a new proxy re-encryption scheme that integrates the SM2 algorithm and PRE+ features has become the key to solving the problem of secure data sharing in IoMT-enabled cloud-based healthcare information systems.

### 1.3. Research Contributions

This paper proposes an SM2-based PRE+ scheme designed for IoMT-enabled cloud-based healthcare information systems. The main contributions are as follows:

1. Constructing a PRE+ scheme suitable for secure sharing of IoMT-based healthcare data. Instead of trivially porting existing PRE+ onto SM2, we redesign the cryptographic structure to satisfy IoMT sensor data scenarios, realizing controlled sharing of medical information while protecting the privacy of sensor-generated healthcare data.
2. Designing an innovative dual-layer architecture of Session Key (SK) and Encapsulation Key (EK), breaking the “one-message-one-public-key-encapsulation” limitation of traditional pairing-free PRE+ schemes. The reusable SK supports multiple medical records within its validity period, reducing repeated public-key cryptographic overhead for resource-constrained IoMT terminals; the EK is dynamically derived per-ciphertext to guarantee forward security. This key-reuse mechanism is absent in prior pairing-free PRE/PRE+ works [10,41,42].
3. The re-encryption key $rk_{A\to B}=r\left(a+b\right)G$ is cryptographically bound to the per-message random scalar *r* and recipient’s public key without additional zero-knowledge proofs. It eliminates the risk of unauthorized transfer of access rights and supports message-level access control, allowing patients to selectively share specific IoMT-generated medical records with different doctors.
4. Abandoning bilinear pairing operations, SM2 elliptic-curve arithmetic significantly reduces computational costs: re-encryption only requires one point-addition operation, and ciphertext expansion is limited to one elliptic-curve point (256 bits), which fits wearable IoMT medical terminals.
5. Formal security proofs are provided for both original ciphertext and transformed re-encrypted ciphertext under the DDH assumption within the Random Oracle Model. Through key isolation and identity-binding design, the scheme achieves collusion resistance and non-transferability. Our implementation adopts the national commercial cryptographic suite SM2 + SM4 for medical-system compliance.

### 1.4. Paper Structure

The rest of this paper is structured as follows. Section 2 gives preliminaries covering the PRE+ paradigm, SM2 algorithm and the DDH problem. Section 3 describes the system and security models for IoMT healthcare data sharing. Section 4 details the six core algorithms of the proposed SM2-PRE+ scheme. Section 5 provides formal security analysis including correctness and security proofs. Section 6 evaluates the functional, computational and storage performance of our scheme. Section 7 draws the conclusion and discusses future work.

## 2. Preliminaries

### 2.1. PRE+

Although traditional Proxy Re-Encryption (PRE) schemes can effectively realize secure delegation of decryption capabilities, they inherently have risks of delegation leakage and transitive re-encryption. Because the re-encryption key is generated by the delegator, it may be reused or abused beyond the original sharing intention. To address these issues, the concept of PRE+ [39] was proposed, which achieves a fundamental paradigm shift in the generation and management of re-encryption keys. In PRE+, the re-encryption key is not generated by the delegator (data owner) but by the encryptor (the entity initiating data encryption). This structural reversal enables the encryptor to have full control over which ciphertexts can be re-encrypted and to whom they can be re-encrypted.

Therefore, PRE+ schemes naturally provide the following key features for secure cloud data sharing:

- Non-transferability: Since the re-encryption key is explicitly bound to the message and the recipient’s public key, neither the proxy nor the recipient can cryptographically delegate decryption capabilities to a third party without the participation of the encryptor [39].
- Message-level access control: Each re-encryption key is bound to a specific ciphertext, supporting selective disclosure of individual medical records rather than batch sharing of all data.
- Revocability and auditability Fine-grained control over each re-encryption operation facilitates accurate permission revocation and detailed logging of access events.

Despite these advantages, existing PRE+ constructions still have limitations in practical deployment, especially in resource-constrained and privacy-sensitive fields such as cloud-assisted healthcare:

- Efficient cryptographic operations: Most existing PRE+ schemes rely heavily on bilinear pairings, which introduce significant computational and storage overhead, making them unsuitable for mobile terminals or embedded devices commonly used in medical systems.
- Lack of key reuse mechanisms: A new re-encryption key must be generated for each message-recipient pair, which exacerbates the complexity of key management in systems handling high-volume data streams (such as continuous health monitoring or remote diagnosis).

It should be emphasized that simply instantiating existing PRE+ logic over SM2 curve cannot achieve our message-bound re-encryption-key property nor the SK/EK dual-layer key-reuse architecture. In [10], the authors present a pairing-free certificate-based PRE+ for general-purpose cloud storage. However, its re-encryption-key is decoupled from per-message randomness, and every message requires complete independent public-key encapsulation without session-key reuse. In [41,42], the schemes are ordinary pairing-free PRE schemes rather than PRE+ paradigm; they cannot guarantee inherent non-transferable authorization. Dedicated structural redesign of cryptographic primitives is required for streaming IoMT sensor scenarios, which forms the core contribution of our SM2-PRE+.

### 2.2. SM2 Algorithm

SM2 Algorithm: The SM2 algorithm is China’s official elliptic curve public-key encryption standard. The full encoding process has four main steps: setting up the system, generating user keys, encoding the plaintext, and decoding the ciphertext. All the elliptic curve operations and parameter calculations used here strictly follow the official national SM2 encryption specs.

### 2.3. Definition of the DDH Problem

Let *G* be a cyclic group under addition with a prime order *q* defined by the SM2 standard, and let *G* be the group generator. Suppose that we have a 4-tuple (G,aG,bG,T), where a and b are unknown numbers in the Zq*. The DDH problem asks whether T=abG. If there is no algorithm that can solve this problem efficiently with any significant advantage, we say that the DDH problem over *G* is hard.

## 3. SM2-PRE+ System Design

### 3.1. System Model for IoMT Healthcare Data Sharing

The end-to-end clinical data-sharing workflow for IoMT is defined as follows.

1. IoMT wearable sensors continuously collect physiological indicators including heart rate and blood pressure and transmit raw sensing data to the trusted patient terminal.
2. The patient terminal encrypts sensor-generated medical records using SM2-PRE+. Only ciphertexts are uploaded to the semi-trusted cloud service provider; plaintext medical data never leaves the patient domain.
3. When remote consultation is required, the patient holds full data ownership and can selectively authorize access for specific medical records to the attending physician, rather than granting full access to all historical health data.
4. The cloud executes proxy re-encryption without accessing plaintext content. The authorized physician downloads transformed ciphertexts and decrypts to obtain medical sensing data for clinical diagnosis.
5. Secondary permission delegation by the authorized physician is cryptographically forbidden, which aligns with real-world clinical requirements.

**Figure 1:**
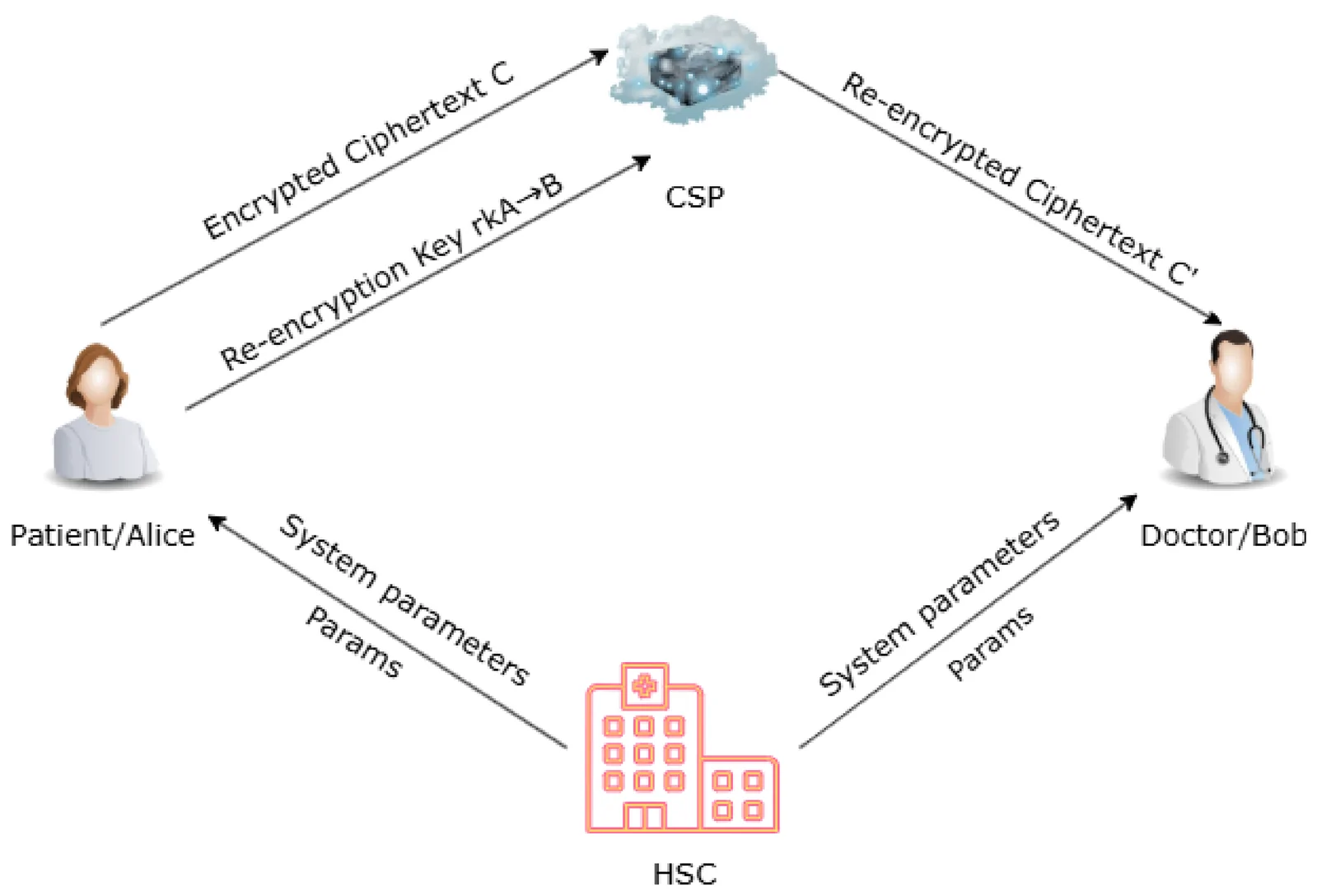
System model.

The system model for the proposed SM2-PRE+ scheme is established for secure medical data sharing in IoMT-enabled healthcare environments. As shown in Figure 1, the proposed framework consists of four entities: Patients (Alice), Doctors (Bob), Cloud Service Providers (CSPs), and Health Service Centers (HSCs). The interactions among these entities enable secure storage and fine-grained sharing of encrypted medical data generated by IoMT devices, while preventing unauthorized access and key abuse.

- Patient (Alice): Generates keys, encrypts medical data collected from IoMT devices, and generates re-encryption keys for authorized doctors.
- Doctor (Bob): Receives re-encrypted ciphertexts and decrypts them using his/her own private key to obtain the original medical data.
- Cloud Service Providers (CSPs): Perform re-encryption based on the key provided by the patient and cannot access the plaintext of the medical data.
- Health Service Center (HSC): Responsible for generating system parameters and making them public.

**Workflow:**

1. Data Generation and Encryption: IoMT devices, such as wearable sensors and medical monitoring terminals, collect healthcare information and transmit it to the patient terminal. The patient encrypts the medical data using SM4 and protects the session key through SM2 encryption.
2. Secure Cloud Storage: Encrypted medical data is uploaded to the cloud. Since only encrypted data is stored, the cloud cannot obtain any sensitive medical information.
3. Authorization and Proxy Re-encryption: When data sharing is required, the patient generates a re-encryption key for an authorized doctor. The cloud server performs ciphertext transformation without learning any sensitive information.
4. Medical Data Recovery: The authorized doctor downloads the transformed ciphertext and uses his/her private key to recover the session key and obtain the original medical data.

By using the above dual-layer key mechanism and lightweight elliptic curve operations, the proposed SM2-PRE+ scheme provides reliable security, efficient computation, and privacy-preserving medical information sharing for resource-constrained IoMT terminals.

Trust assumption for Health Service Center (HSC): HSC acts as a fully-trusted parameter-issuing authority. It generates and publishes global public system parameters params only. HSC never generates, stores, or obtains any user’s private key. Each user generates its own public-private key pair locally on trusted terminal and only uploads the public key to HSC for registration. If HSC becomes compromised, only public parameters could be tampered, and no user private-key material will be leaked.

Two practical deployment modes are supported in our framework. In our baseline architecture, resource-constrained IoMT wearable sensors only collect raw physiological data. They forward these data to the patient-controlled trusted terminal. All SM2 elliptic-curve operations, SM4 encryption-decryption, session-key SK generation, and encapsulation-key EK derivation run on this patient-side trusted terminal. As an optional enhanced mode, powerful smart IoMT terminals can execute the complete ENCRYPT algorithm locally.

### 3.2. Security Model

To ensure the confidentiality and controlled sharing of medical data in cloud environments, the proposed SM2-PRE+ scheme is designed to achieve three core security properties: IND-CCA security, collusion resistance, and non-transferability.

#### 3.2.1. Adversary Model

We consider probabilistic polynomial-time (PPT) adversaries interacting with the system through oracle queries. Two types of adversaries are defined:

- Type-I adversary: models an external attacker or an honest-but-curious cloud service provider (CSP). This adversary does not possess any user private key but may request encryption, re-encryption, and decryption of non-challenge ciphertexts.
- Type-II adversary: models a malicious doctor colluding with the CSP. This adversary may obtain private keys of non-target doctors and corresponding re-encryption keys but never the private key of the target patient.

#### 3.2.2. IND-CCA Security

The secrecy of both the original and re-encrypted ciphertexts is described by the IND-CCA model, which stands for indistinguishability under chosen-ciphertext attack. Here, an attacker can make a bunch of queries to oracles, like generating keys, encrypting, re-encrypting, or decrypting, except for the challenge ciphertext. In the challenge phase, the attacker sends two messages of the same length, and the challenger sends back the encryption of one of them chosen at random. The attacker wins if they can tell which message was encrypted with more than a tiny advantage.

#### 3.2.3. Collusion Resistance

Collusion resistance ensures that even if the CSP colludes with authorized doctors, they cannot derive the patient’s private key or generate valid re-encryption keys for unauthorized users.

Formally, given the private keys of non-target doctors and corresponding re-encryption keys, a PPT adversary should not be able to output either the patient’s private key or a valid re-encryption key for an unauthorized doctor with non-negligible probability.

#### 3.2.4. Non-Transferability

Non-transferability is a fundamental property of PRE+ schemes, ensuring that re-encryption rights cannot be further delegated. Specifically, given a re-encryption key $rk_{A\to B}$ associated with a ciphertext and recipient *B*, neither the proxy nor the recipient can derive a valid re-encryption key $rk_{A\to C}$ for another user *C* or for a different ciphertext without the participation of the original encryptor.

#### 3.2.5. Healthcare-Specific Threats

Beyond general cryptographic adversaries, we define domain-specific threats for IoMT medical scenarios:

- Threat-H1: A semi-trusted cloud service provider attempts to infer sensitive physiological information from ciphertexts collected by wearable IoMT sensors.
- Threat-H2: Authorized medical staff collude with cloud proxies to illegally transfer access permissions to third-party personnel (secondary-delegation attack).
- Threat-H3: Malicious adversaries replay historical IoMT ciphertexts to mislead clinical diagnosis decisions.
- Threat-H4: Malicious insiders inside medical institutions attempt to obtain unauthorized patients’ private health records.

## 4. PRE+ Secure Sharing Scheme for IoMT Healthcare Data Sharing

This section presents the proposed SM2-based PRE+ scheme for secure medical data sharing in cloud-assisted IoMT environments. The scheme consists of six polynomial-time algorithms: Setup, KeyGen, Encrypt, ReKeyGen, ReEncrypt, and Decrypt.

**Figure 2:**
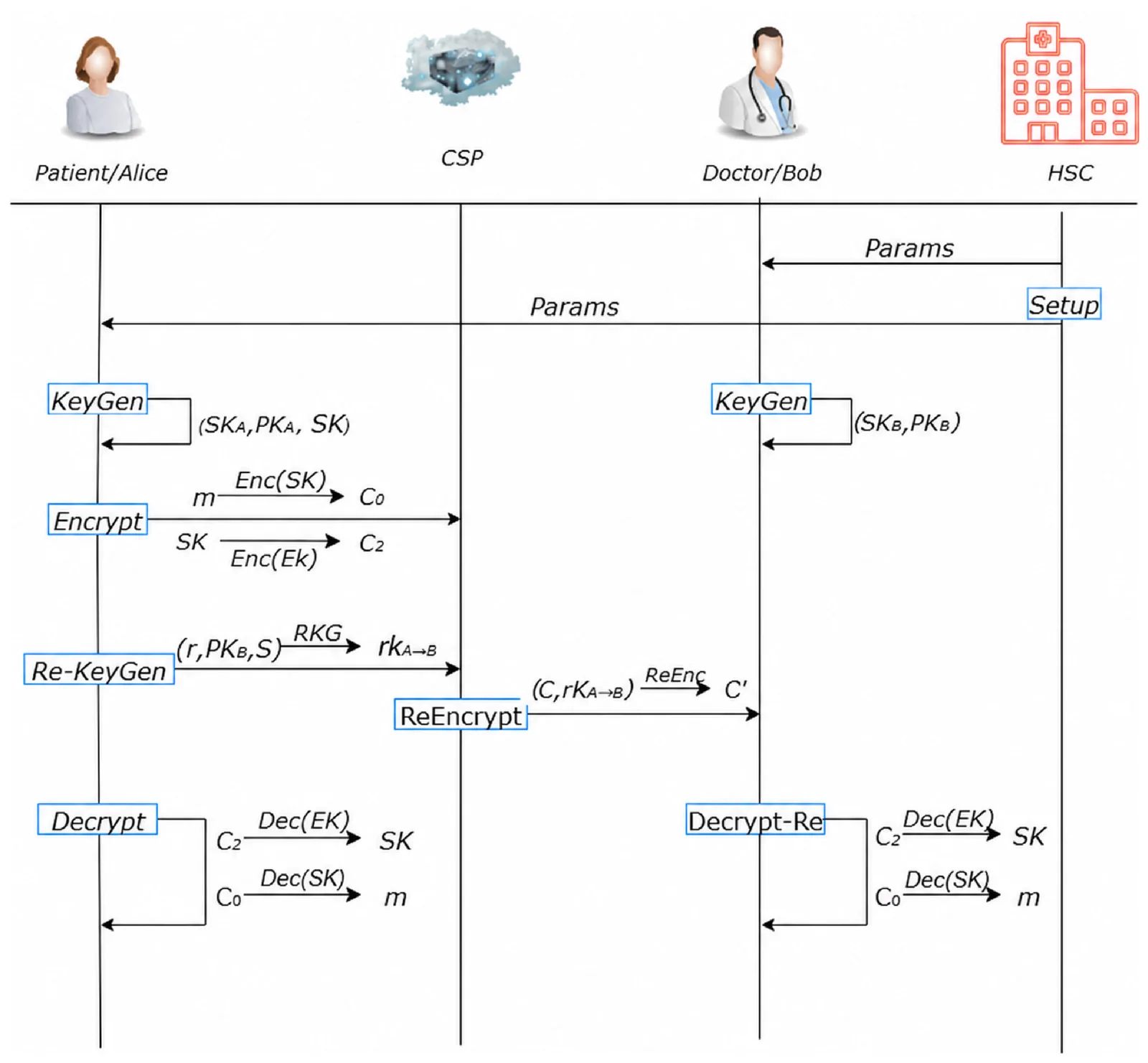
Scheme flow chart.

The overall workflow is illustrated in Figure 2, and the key notations used throughout the scheme are summarized in Table 1.

### 4.1. Setup

The Setup algorithm is executed by the Health Service Center (HSC) to initialize the system. Given a security parameter λ, the HSC selects a cyclic additive group *G* of prime order *q* with generator *G* as specified by the SM2 standard. Three cryptographic hash functions are defined:

$$H_{1}:{\left\{0,1\right\}}^{*}\to G,\;H_{2}:G\to {\left\{0,1\right\}}^{k},\;H_{3}:{\left\{0,1\right\}}^{*}\to Z_{q}.$$

where parameter *k* denotes the bit-length of SM4 symmetric key, i.e., 128 bits. Particularly, hash function $H_{2}$ takes an elliptic-curve group element from group *G* as its input and outputs a fixed-length *k*-bit binary string ${\left\{0,1\right\}}^{k}$. SM4 is adopted as the symmetric encryption algorithm, and *T* denotes the default validity period of the session key. The system public parameters are as follows:

$$params=(G,q,G,H_{1},H_{2},H_{3},SM_{4},T).$$

### 4.2. Key Generation

The KEYGEN algorithm is executed locally by each entity on its trusted terminal to generate public–private key pairs. For patient A and doctor B, each user randomly selects secret values $a,b\overset{R}{\leftarrow }Z_{q}^{*}$ locally and computes the following:

$$\left(pk_{A},sk_{A}\right)=\left(aG,a\right),\;\left(pk_{B},sk_{B}\right)=\left(bG,b\right).$$

Additionally, patient A pre-derives a long-term session key SKfor the current validity period as follows:

$$SK=H_{2}(a\cdot H_{1}\left(ID_{A}∥t_{0})\right),$$

where $t_{0}$ is the timestamp indicating the start of the validity period. This SK will be reused for encrypting multiple medical records within the time window $\left[t_{0},t_{0}+T\right]$.

Security justification for session-key reuse: Although SK is reused for multiple medical records within validity window $\left[t_{0},t_{0}+T\right]$, each ciphertext generates an independent fresh encapsulation key $EK=H_{1}\left(r\cdot pk_{A}\right)$ using per-message randomness *r*. The session key SK is wrapped by distinct EK in each ciphertext via $C_{2}=SM4_{EK}\left(SK\right)$. Breach of a single EK cannot reveal SK. SK is time-bound and must be regenerated upon timestamp expiration *T*. The hash-derived SK maintains pseudorandomness under the Random Oracle Model for repeated encryption.

### 4.3. Encryption

The ENCRYPT algorithm is executed by the patient-trusted terminal (baseline architecture), or optionally by capable IoMT terminal, to encrypt a medical message M. Given the patient’s public key $pk_{A}$ and the pre-derived session key SK, the terminal chooses a random $r\in Z_{q}^{*}$ and computes the following:

$$C_{1}=rG,$$

$$K=r\cdot pk_{A}=raG,$$

$$EK=H_{1}\left(K\right).$$

The ciphertext components are then generated as follows:

$$C_{0}=SM4_{SK}\left(M\right),\;C_{2}=SM4_{EK}\left(SK\right),$$

$$C_{3}=H_{3}(C_{0}∥C_{1}∥C_{2}∥pk_{A}∥t),$$

where *t* is the current timestamp. The final ciphertext is as follows:

$$C=(C_{0},C_{1},C_{2},C_{3}).$$

Note on randomness r: The value $C_{1}=rG$ is a public elliptic-curve point uploaded to cloud storage. The scalar random value $r\in Z_{q}^{*}$ cannot be recovered from public point $C_{1}=rG$, since extracting scalar from a group point is equivalent to solving the ECDLP problem which is computationally hard. The raw scalar *r* must be persistently stored locally within the patient’s trusted terminal (secure element or mobile secure sandbox), indexed by ciphertext ID and timestamp. The scalar *r* is never transmitted to the cloud server.

### 4.4. Re-Encryption Key Generation

The ReKeyGen algorithm is executed by the patient *A* to delegate decryption rights to the doctor *B*. Using the same random value *r* from the encryption phase and the doctor’s public key $pk_{B}$, the patient computes the following:

$$S=r\left(sk_{A}\cdot G+pk_{B}\right)=r\left(aG+bG\right)=r\left(a+b\right)G,$$

and outputs the re-encryption key:

$$rk_{A\to B}=S.$$

This key is securely transmitted from the patient to the cloud proxy.

### 4.5. Re-Encryption

The ReEncrypt algorithm is executed by the cloud proxy to transform a ciphertext intended for the patient into one decryptable by the authorized doctor. Upon receiving $C=(C_{0},C_{1},C_{2},C_{3})$ and the re-encryption key $rk_{A\to B}=S$, the proxy first verifies the integrity tag:

$$H_{3}(C_{0}∥C_{1}∥C_{2}∥pk_{A}∥t)\overset{?}{=}C_{3}.$$

If the verification fails, the proxy outputs ⊥. Otherwise, the re-encrypted ciphertext is constructed as follows:

$$C^{\prime }=\left(C_{0},C_{1},C_{2},C_{3},D\right),$$

where D=S.

For long-term cloud-archived medical records, the patient retrieves the locally stored scalar *r* by the corresponding ciphertext identifier before calling ReKeyGen. One practical limitation should be noted. If the patient-side local storage of scalar *r* for a given ciphertext is lost, valid re-encryption keys for that ciphertext cannot be produced anymore.

### 4.6. Decryption

The Decrypt algorithm is executed by the legitimate recipient to recover the plaintext *M*.

#### 4.6.1. Patient Decryption

For the original ciphertext $C=(C_{0},C_{1},C_{2},C_{3})$, the patient *A* uses $sk_{A}=a$ to compute the following:

$$K=a\cdot C_{1}=raG,$$

$$EK=H_{1}\left(K\right),\;SK=SM4_{EK}^{-1}\left(C_{2}\right),\;M=SM4_{SK}^{-1}\left(C_{0}\right).$$

The integrity tag is verified by checking $C_{3}=H_{3}(C_{0}∥C_{1}∥C_{2}∥pk_{A}∥t)$. If valid, the patient outputs *M*.

#### 4.6.2. Doctor Decryption

For the re-encrypted ciphertext $C^{\prime }=\left(C_{0},C_{1},C_{2},C_{3},D\right)$, the doctor *B* uses $sk_{B}=b$ to compute the following:

$$K=D-b\cdot C_{1}=r\left(a+b\right)G-b\cdot rG=raG.$$

Then, the doctor derives $EK=H_{1}\left(K\right)$, recovers $SK=SM4_{EK}^{-1}\left(C_{2}\right)$, and obtains $M=SM4_{SK}^{-1}\left(C_{0}\right)$. The same integrity verification on $C_{3}$ is performed before outputting *M*.

## 5. Security Analysis

In this part, we look at how secure the proposed proxy re-encryption scheme is. The analysis shows that the scheme works correctly, IND-CCA confidentiality, re-encrypted ciphertext security, collusion resistance, and non-transferability under standard cryptographic assumptions.

### 5.1. Correctness

Decryption of original ciphertext:

$$K^{\prime }=sk_{A}\cdot C_{1}=sk_{A}\cdot r\cdot G=r\cdot sk_{A}\cdot G=K.$$

$$EK^{\prime }=H_{1}\left(K^{\prime }\right)=EK$$

$$SK^{\prime }=SM4_{EK^{\prime }}^{-1}\left(C_{2}\right)=SM4_{EK}^{-1}\left(C_{2}\right)$$

$$SM4_{SK^{\prime }}^{-1}\left(C_{0}\right)=SM4_{SK}^{-1}\left(C_{0}\right)=M$$

Decryption of re-encrypted ciphertext:

$$K^{\prime \prime }=D-b\cdot C_{1}^{\prime }=r\left(a+b\right)G-brG=raG=K.$$

$$EK^{\prime \prime }=H_{1}\left(K^{\prime \prime }\right)=EK$$

$$SK^{\prime \prime }=SM4_{EK^{\prime \prime }}^{-1}\left(C_{2}^{\prime }\right)=SM4_{EK}^{-1}\left(C_{2}^{\prime }\right)$$

$$SM4_{SK^{\prime \prime }}^{-1}\left(C_{0}^{\prime }\right)=SM4_{SK}^{-1}\left(C_{0}^{\prime }\right)=M$$

### 5.2. IND-CCA Security of Original Ciphertexts

**Theorem 1.** 
*Under the DDH assumption in group G, the proposed scheme achieves IND-CCA security for original ciphertexts in the random oracle model.*

**Proof.**  Suppose that there exists a probabilistic polynomial-time adversary A that can break the IND-CCA security of the proposed encryption scheme with non-negligible advantage ϵ. We construct a simulator S that uses A to solve the DDH problem.

Given a DDH instance (G,aG,bG,Z), the simulator must determine whether Z=abG. The simulator does not know the discrete logarithms *a* or *b*.

Setup The simulator sets the target (challenge) public key as

$$pk_{A}^{*}=aG.$$

Hash functions $H_{1},H_{2},H_{3}$ are modeled as random oracles. The simulator initializes all oracle-tracking lists, all initially empty: $L_{1}$ for $H_{1}$, $L_{2}$ for $H_{2}$, $L_{3}$ for $H_{3}$, $L_{sk}$ for user private-key records, $L_{r}$ for ephemeral randomness *r* of simulator-generated ciphertexts, $L_{cipher}$ to store plaintext/intermediate values for simulator-generated ciphertexts.

For each user identity $ID_{i}$, if $ID_{i}$ is not the target identity $ID_{A}^{*}$, the simulator generates the key material locally; for target $ID_{A}^{*}$, only the public key $pk_{A}^{*}=aG$ is known, and the corresponding private key *a* remains unknown.

Remark on oracle interface: Different from conventional PRE, our PRE+ re-encryption key $rk_{A\to B}$ is message-bound and depends on per-ciphertext random scalar *r*. Thus, the re-encryption-key oracle takes $\left(ID_{A},ID_{B},C\right)$ as input, where *C* denotes the target ciphertext associated with randomness *r*, instead of only identity pair $\left(ID_{A},ID_{B}\right)$.

Hash Oracle Simulation.

For $H_{1}$ queries on input x∈G, the simulator checks if $\left(x,h_{1}\right)$ exists in $L_{1}$. If so, return $h_{1}$. Otherwise, sample uniformly random $h_{1}\in G$, store $\left(x,h_{1}\right)$ in $L_{1}$, and return $h_{1}$.

For $H_{2}$ queries on input X∈G, check if $\left(X,h_{2}\right)$ exists in $L_{2}$. If so, return $h_{2}$. Otherwise sample $h_{2}\in {\left\{0,1\right\}}^{k}$, store $\left(X,h_{2}\right)$ in $L_{2}$, and return $h_{2}$.

For $H_{3}$ queries on input $\left(C_{0}∥C_{1}∥C_{2}∥pk∥t\right)$, check if tuple exists in $L_{3}$. If it exists return the stored value. Otherwise sample $h_{3}\in Z_{q}^{*}$, store the tuple and $h_{3}$ in $L_{3}$, and return $h_{3}$.

Key Generation Oracle Simulation.

- If $ID_{i}=ID_{A}^{*}$ (target patient), return the public key $pk_{A}^{*}=aG$, and the simulator holds no corresponding private key.
- For other identity $ID_{i}$, sample $sk_{i}\overset{R}{\leftarrow }Z_{q}^{*}$, compute $pk_{i}=sk_{i}G$, store $\left(ID_{i},sk_{i},pk_{i}\right)$ in $L_{sk}$, and return $pk_{i}$.

Re-Encryption Key Generation Oracle Simulation.

Input: $\left(ID_{A},ID_{B},C\right)$, ciphertext *C* with component $C_{1}=rG$.

- If $ID_{A}\neq ID_{A}^{*}$, the simulator retrieves the known $sk_{A}$ from $L_{sk}$, generates genuine $rk_{A\to B}$ following scheme specification, and returns $rk_{A\to B}$.
- If $ID_{A}=ID_{A}^{*}$ (target user), conduct the following:
  1. Check whether ciphertext *C* was generated by the simulator, and look-up $L_{r}$ to retrieve corresponding ephemeral *r*.
  2. If *r* is available from $L_{r}$, compute
    $$rk_{A^{*}\to B}=r\left(pk_{A}^{*}+pk_{B}\right)=r\left(aG+pk_{B}\right).$$
    Since $pk_{A}^{*}=aG$, this expression is algebraically identical to real $rk_{real}=r\left(sk_{A}^{*}G+pk_{B}\right)$. The simulated re-encryption key is perfectly indistinguishable from the honestly generated key, and the following is returned: $rk_{A^{*}\to B}$.
  3. If ciphertext *C* is adversary-generated and *r* is unknown to simulator, the simulator cannot reconstruct the valid message-bound re-encryption key for this adversary-supplied ciphertext, and the following is returned: ⊥.

Re-Encryption Oracle Simulation.

Input: ciphertext *C*, target re-encryption-key $rk_{A\to B}$. Under the IND-CCA game rule, re-encryption queries on the challenge ciphertext after the challenge phase are strictly forbidden and shall be rejected.

- If *C* is a ciphertext generated by the simulator and the valid $rk_{A\to B}$ is retrieved from oracle records, perform the honest ReEncrypt procedure. First, verify integrity tag $C_{3}$. If verification passes, construct and return the transformed re-encrypted ciphertext $C^{\prime }$. If the integrity check fails, return ⊥.
- If *C* is supplied by the adversary, first check the integrity tag via entry lookup in the random-oracle list $L_{3}$. Return ⊥ if the integrity verification fails. If integrity holds but the corresponding ephemeral randomness *r* for *C* is unknown to the simulator, return ⊥. The simulator cannot generate valid re-encrypted output without knowing the per-message random scalar *r*.

Decryption Oracle Simulation. The simulator answers decryption queries without accessing target private key $sk_{A}^{*}=a$.

Input: ciphertext $C=(C_{0},C_{1},C_{2},C_{3})$, public key pk.

1. Case 1: $pk\neq pk_{A}^{*}$. The simulator fetches the corresponding private key from stored list $L_{sk}$. It runs the genuine decryption algorithm and returns recovered plaintext *M*.
2. Case 2: $pk=pk_{A}^{*}$ (target public key, *a* remains unknown).
  (a) Integrity check: look up tuple $\left(C_{0}∥C_{1}∥C_{2}∥pk_{A}^{*}∥t\right)$ in random-oracle list $L_{3}$. Return ⊥ if no matching entry exists.
  (b) If ciphertext *C* was produced by the simulator and recorded in $L_{cipher}$, directly return the stored plaintext *M*.
  (c) If *C* is adversary-supplied and no matching entry can be found among random-oracle lists $L_{1}$, return ⊥.
  (d) Under Random Oracle Model, the adversary can produce a valid fresh ciphertext without corresponding random-oracle queries only by guessing hash outputs or breaking the SM4 symmetric cipher. This event yields negligible success probability bounded by $\frac{q_{H}}{2^{k}}+Adv_{SM4}$.

Challenge Phase. The adversary submits two equal-length messages $\left(M_{0},M_{1}\right)$. The simulator samples $\beta \overset{R}{\leftarrow }\left\{0,1\right\}$. Recall that in our scheme, session-key SK is derived from $SK=H_{2}\left(a\cdot H_{1}\left(ID_{A}^{*}∥t_{0}\right)\right)$, independent of challenge ephemeral randomness. Take DDH instance element $C_{1}^{*}=bG$, and set the DH value $K^{*}=Z$. Compute the encapsulation key $EK^{*}=H_{1}\left(K^{*}\right)$. Construct the challenge ciphertext components:

$$C_{0}^{*}=SM4_{SK}\left(M_{\beta }\right),\;C_{2}^{*}=SM4_{EK^{*}}\left(SK\right),\;C_{3}^{*}=H_{3}\left(C_{0}^{*}∥C_{1}^{*}∥C_{2}^{*}∥pk_{A}^{*}∥t^{*}\right).$$

The challenge ciphertext is $C^{*}=\left(C_{0}^{*},C_{1}^{*},C_{2}^{*},C_{3}^{*}\right)$.

If Z=abG, then $K^{*}=abG=b\cdot pk_{A}^{*}$ matches the genuine encryption distribution with ephemeral r=b. If *Z* is a random group element, $K^{*}$ is statistically independent of $pk_{A}^{*}$, and the challenge carries no information about $M_{\beta }$.

Analysis. For re-encryption-key queries against simulator-generated ciphertexts, the simulated $rk_{sim}=r\left(pk_{A}^{*}+pk_{B}\right)$ is algebraically equal to the real $rk_{real}=r\left(sk_{A}^{*}G+pk_{B}\right)$, giving a perfect simulation.

For decryption oracle responses, the adversary can make the simulator fail only by forging valid ciphertext without issuing corresponding random-oracle queries. Let $q_{H}$ denote the maximum number of random-oracle queries made by the adversary. This failure probability is bounded by $\frac{q_{H}}{2^{k}}+Adv_{SM4}$.

If the adversary A distinguishes the challenge bit β with the advantage ϵ, then the simulator S solves the DDH problem with the same advantage. We obtain the following bound:

$$Adv_{A}^{\mathrm{IND}\text{-}\mathrm{CCA}}\leq Adv_{B}^{\mathrm{DDH}}+\frac{q_{H}}{2^{k}}+Adv_{SM4}+negl\left(\lambda \right).$$

Under the DDH hardness assumption and secure SM4 symmetric cipher, the adversary’s advantage is negligible. The scheme satisfies IND-CCA security for the original ciphertext. □

### 5.3. IND-CCA Security of Re-Encrypted Ciphertexts

**Lemma 1.** 
*For a type-II attacker going after re-encrypted ciphertexts, our proposed scheme is IND-CCA secure under the DDH assumption.*

**Proof.**  Assume that an adversary A can break the confidentiality of re-encrypted ciphertexts with non-negligible advantage. We construct a simulator S to solve the DDH problem.

Given a DDH instance (G,aG,bG,Z), the simulator sets the delegatee’s public key as

$$pk_{B}^{*}=aG.$$

For the delegator *A*, the simulator selects a random private key $sk_{A}\in Z_{q}^{*}$ and computes

$$pk_{A}=sk_{A}G.$$

Query Phase.

Re-Encryption-Key Queries: For query $\left(ID_{A},ID_{B}^{*},C\right)$, the simulator generates a simulated re-encryption key which is computationally indistinguishable from honestly generated real keys.

Re-Encryption Oracle Queries: Given ciphertext *C* and the corresponding re-encryption key, the simulator first validates the integrity tag from list $L_{3}$.

- If *C* is simulator-generated with known ephemeral randomness *r*, perform the honest re-encryption algorithm and output the transformed ciphertext $C^{\prime }$.
- If *C* is adversary-provided and the ephemeral randomness *r* is unknown to simulator, return ⊥.
- Re-encryption queries for challenge-derived ciphertexts after the challenge phase are strictly forbidden and return ⊥.

Decryption-Re-Oracle Queries: This oracle handles decryption for already-transformed re-encrypted ciphertext $C^{\prime }$.

- If $C^{\prime }$ originates from simulator-generated ciphertext, verify integrity via $L_{3}$ entries and return stored plaintext.
- If $C^{\prime }$ is adversary-supplied without matching random-oracle records, return ⊥.
- Decryption-Re queries targeting challenge-derived ciphertexts after the challenge phase are disallowed and return ⊥.

Challenge Phase. The adversary submits two messages $\left(M_{0},M_{1}\right)$. The simulator randomly selects β∈{0,1} and constructs the challenge ciphertext as

$$C_{1}^{\prime \prime }=bG$$

$$D^{*}=Z+sk_{A}\left(bG\right).$$

IfZ=abG,then

$$D^{*}=abG+sk_{A}bG=b\left(a+sk_{A}\right)G,$$

which matches the distribution of a valid re-encrypted ciphertext. Otherwise, $D^{*}$ is random in group *G*.

Analysis.Therefore, if the opponent has an edge of ϵ in telling the challenge ciphertext apart, the simulator could solve the DDH problem with the same edge, which goes against the DDH assumption. Hence, the proposed scheme achieves IND-CCA security for re-encrypted ciphertexts. □

### 5.4. Proof of Collusion Resistance

**Theorem 2.** 
*Under the Random Oracle Model (ROM), if the Elliptic Curve Discrete Logarithm Problem (ECDLP) over group G is computationally hard, the proposed scheme satisfies the collusion resistance property.*

**Proof.**  The re-encryption key from user *A* to *B* is

$$rk_{A\to B}=r\left(sk_{A}G+pk_{B}\right).$$

Even if the proxy colludes with doctor B, they can compute $rk_{A\to B}-b\cdot pk_{A}=rsk_{A}G$. Recovering the secret value $sk_{A}$ from the group element $rsk_{A}G$ requires solving the elliptic-curve discrete logarithm problem. This problem is computationally infeasible under current computing capabilities. Thus, the scheme satisfies collusion-resistance. □

### 5.5. Proof of Non-Transferability

**Theorem 3.** 
*Under the Random Oracle Model (ROM), if the Elliptic Curve Discrete Logarithm Problem (ECDLP) over group G is computationally hard, the proposed scheme satisfies the non-transferability property.*

**Proof.**  To transfer the delegation from *B* to another user *C*, a valid re-encryption key

$$rk_{A\to C}=r\left(sk_{A}G+pk_{C}\right)$$

must be generated. However, this requires knowledge of the delegator’s private key $sk_{A}$. Since deriving $sk_{A}$ from $pk_{A}=sk_{A}G$ requires solving the ECDLP, which is infeasible, the delegation capability cannot be transferred to other users. □

## 6. Performance Analysis

To quantitatively compare the performance of the proposed scheme (hereinafter referred to as “our scheme”) with the reference schemes [21,22,29,41,42], we measure and analyze from two levels: measured primitive time consumption (platform micro-benchmarks) and normalized time estimation based on the primitive counts of high-level operations provided in this papers. This also allows comparing “algorithmic complexity differences” in the same implementation environment and minimizing implementation differences. The experiments were conducted on a laptop with an Intel(R) Core(TM) i5-8300H CPU @ 2.30 GHz processor and 8.00 GB of memory, using Python 3.8.0. The 256-bit prime field elliptic curve recommended in the SM2 elliptic curve public key cryptography algorithm standard was adopted, i.e., $y^{2}=x^{3}+ax+b$, with the curve parameters shown in Table 2.

### 6.1. Scheme Comparison

In terms of functional comparison, the SM2-based PRE+ scheme proposed in this paper differs from existing works in the following dimensions. Compared with Zhou et al.’s pairing-free PRE scheme [41] designed for cloud medical information systems, our scheme also achieves pairing-free implementation and provides collusion resistance and CCA security proof. However, [41] is a conventional PRE construction rather than a PRE+ paradigm—it does not provide non-transferability of re-encryption keys, nor does it employ a session-key reuse mechanism to reduce repeated public-key operations for high-frequency IoMT data streams. In addition, compared with the PF-PREET proposed by Han [42], this work explicitly provides the definition and proof of IND-sID-CCA security and supports multi-level authorization for equality-test; however, it does not claim non-transferability. Different from prior pairing-free PRE/PRE+ [10,41,42], our SM2-PRE+ realizes two new cryptographic primitives: message-bound re-encryption-key construction and SK/EK dual-layer reusable key architecture, together with complete IND-CCA proof covering both original and re-encrypted ciphertext.The functional features of different schemes are summarized in Table 3.

In summary, the advantages of the proposed scheme are reflected in the following: (1) compliant implementation of the national cryptographic SM2 algorithm and session key reuse, reducing the overhead of mobile medical terminals; (2) joint design of non-transferability and fine-grained message-level authorization, enhancing the principle of least privilege and abuse prevention in cloud-assisted IoMT healthcare system sharing scenarios.

### 6.2. Computational Overhead

The computational overhead comparison between the SM2-based PRE+ scheme proposed in this paper (hereinafter referred to as “our scheme”) and the reference schemes [22,29,41,42] is mainly based on the sum of primitive operation counts (elliptic curve scalar multiplication $S_{G}$, point addition $A_{G}$, and hash *H*). We first performed micro-benchmark measurements on the most important primitives (5000 samples for each item): elliptic curve scalar multiplication (ScalarMul, denoted as $t_{G}$), point addition (PointAdd, denoted as $t_{A}$), and hash (Hash, denoted as $t_{H}$). The measured average time consumption of the primitives was used for subsequent normalized estimation. For any high-level operation (such as Encrypt, ReKeyGen, ReEncrypt, and Decrypt), if the operation consists of $n_{G}$ scalar multiplications, $n_{A}$ point additions, and $n_{H}$ hashes, the theoretical execution time estimation on the target platform is $T=n_{G}t_{G}+n_{A}t_{A}+n_{H}t_{H}$. We substituted the primitive counts provided in the reference papers into the above formula to obtain the theoretical time estimation of the two schemes under the same platform and implementation conditions (see Table 4). Note that some reference schemes rely on special pairing-friendly curves and cannot be ported directly to our SM2 curve. In such cases, the reported runtime is a normalized estimation. It predicts the runtime when the corresponding algorithm runs on our experimental platform. This estimation removes most platform-dependent deviations. Still, results can be influenced by crypto-library differences, constant overheads, and memory-access behaviors. Therefore, we also provide the primitive complexity (number of G/A/H operations) in the discussion to facilitate readers to compare the pure algorithm-level complexity differences.

Note: The total runtime reported in this section does not include SM4 encryption and decryption overhead. For hybrid proxy re-encryption constructions, symmetric ciphers are widely adopted across competing schemes for payload encryption. Following common evaluation conventions in the PRE-related literature, we exclude symmetric-cipher overhead and focus the comparison on core proxy-re-encryption-related asymmetric operations. This treatment ensures fair cross-scheme comparison and does not change the relative performance ranking among evaluated schemes.

In the proposed SM2-PRE+ scheme, the time consumption of each phase is as follows:

- Key Generation (KeyGen): $2t_{G}+2t_{H}=2\times 6.887+2\times 0.001=13.776\;\mathrm{ms}$;
- Encryption (Encrypt): $2t_{G}+2t_{H}=2\times 6.887+2\times 0.001=13.776\;\mathrm{ms}$;
- Re-encryption Key Generation (ReKeyGen): $1t_{G}+1t_{A}=6.887+0.000=6.887\;\mathrm{ms}$;
- Re-encryption (ReEnc): $1t_{A}+1t_{H}=0.000+0.001=0.001\;\mathrm{ms}$;
- Decryption (Decrypt): $1t_{G}+2t_{H}=6.887+2\times 0.001=6.889\;\mathrm{ms}$;
- Re-decryption (ReDecrypt): $1t_{G}+1t_{A}+2t_{H}=6.887+0.000+2\times 0.001=6.889\;\mathrm{ms}$.

The total computational cost of the proposed SM2-PRE+ scheme is 13.776+13.776+6.887+0.001+6.889+6.889=48.218ms.

In the scheme [41], the time consumption of each phase is as follows:

- Key Generation (KeyGen): $2t_{G}=2\times 6.887=13.774\;\mathrm{ms}$;
- Encryption (Encrypt): $3t_{G}+1t_{A}+1t_{H}=3\times 6.887+0.000+0.001=20.662\;\mathrm{ms}$;
- Re-encryption Key Generation (ReKeyGen): $1t_{G}+1t_{H}=6.887+0.001=6.888\;\mathrm{ms}$;
- Re-encryption (ReEnc): $5t_{G}+3t_{A}+3t_{H}=5\times 6.887+3\times 0.000+3\times 0.001=34.438\;\mathrm{ms}$;
- Decryption (Decrypt): $3t_{G}+2t_{H}=3\times 6.887+2\times 0.001=20.663\;\mathrm{ms}$;
- Re-decryption (ReDecrypt): $4t_{G}+3t_{H}=4\times 6.887+3\times 0.001=27.551\;\mathrm{ms}$.

The total computational cost of the scheme [41] is 13.774+20.662+6.888+34.438+20.663+27.551=123.976ms.

In the scheme [29], the time consumption of each phase is as follows:

- Key Generation (KeyGen): $2t_{G}+1t_{H}=2\times 6.887+0.001=13.775\;\mathrm{ms}$;
- Encryption (Encrypt): $2t_{G}+2t_{A}+3t_{H}=2\times 6.887+2\times 0.000+3\times 0.001=13.777\;\mathrm{ms}$;
- Re-encryption Key Generation (ReKeyGen): $2t_{G}+3t_{H}=2\times 6.887+3\times 0.001=13.777\;\mathrm{ms}$;
- Re-encryption (ReEnc): $1t_{G}+1t_{H}=6.887+0.001=6.888\;\mathrm{ms}$;
- Decryption (Decrypt): $1t_{G}+2t_{H}=6.887+2\times 0.001=6.889\;\mathrm{ms}$;
- Re-decryption (ReDecrypt): $2t_{G}+3t_{H}=2\times 6.887+3\times 0.001=13.777\;\mathrm{ms}$.

The total computational cost of the scheme [29] is 13.775+13.777+13.777+6.888+6.889+13.777=68.883ms.

In the scheme [42], the time consumption of each phase is as follows:

- Key Generation (KeyGen): $2t_{G}=2\times 6.887=13.774\;\mathrm{ms}$;
- Encryption (Encrypt): $5t_{G}+2t_{H}=5\times 6.887+2\times 0.001=34.437\;\mathrm{ms}$;
- Re-encryption Key Generation (ReKeyGen): $2t_{G}+1t_{A}=2\times 6.887+0.000=13.774\;\mathrm{ms}$;
- Re-encryption (ReEnc): $1t_{G}+1t_{H}=6.887+0.001=6.888\;\mathrm{ms}$;
- Decryption (Decrypt): $2t_{G}+2t_{H}=2\times 6.887+2\times 0.001=13.776\;\mathrm{ms}$;
- Re-decryption (ReDecrypt): $2t_{G}+1t_{H}=2\times 6.887+0.001=13.775\;\mathrm{ms}$.

The total computational cost of the scheme [42] is 13.774+34.437+13.774+6.888+13.776+13.775=96.424ms.

In the scheme [22], the time consumption of each phase is as follows:

- Key Generation (KeyGen): $2t_{G}+2t_{H}=2\times 6.887+2\times 0.001=13.776\;\mathrm{ms}$;
- Encryption (Encrypt): $1t_{G}+4t_{H}=6.887+4\times 0.001=6.891\;\mathrm{ms}$;
- Re-encryption Key Generation (ReKeyGen): $1t_{G}+3t_{H}=6.887+3\times 0.001=6.890\;\mathrm{ms}$;
- Re-encryption (ReEnc): $1t_{G}=6.887\;\mathrm{ms}$;
- Decryption (Decrypt): $1t_{G}+1t_{H}=6.887+0.001=6.888\;\mathrm{ms}$;
- Re-decryption (ReDecrypt): $1t_{G}+5t_{H}=6.887+5\times 0.001=6.892\;\mathrm{ms}$.

The total computational cost of the scheme [22] is 13.776+6.891+6.890+6.887+6.888+6.892=48.324ms.

**Figure 3:**
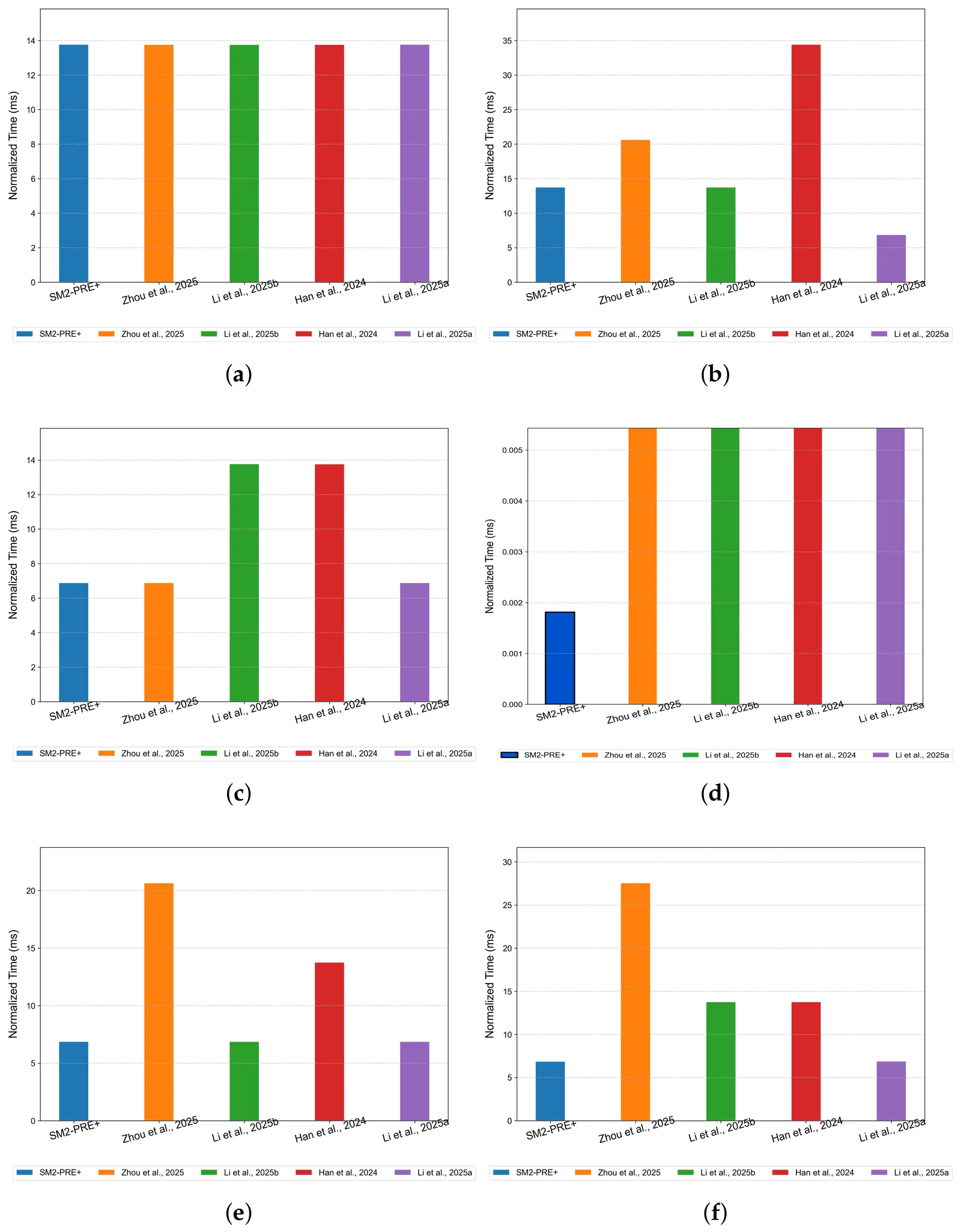
(**a**) Computational overhead of key generation. (**b**) Computational overhead of encryption. (**c**) Computational overhead of re-key generation. (**d**) Computational overhead of re-encryption. (**e**) Computational overhead of decryption. (**f**) Computational overhead of re-decryption.The compared competing schemes are from [22,29,41,42].

**Figure 4:**
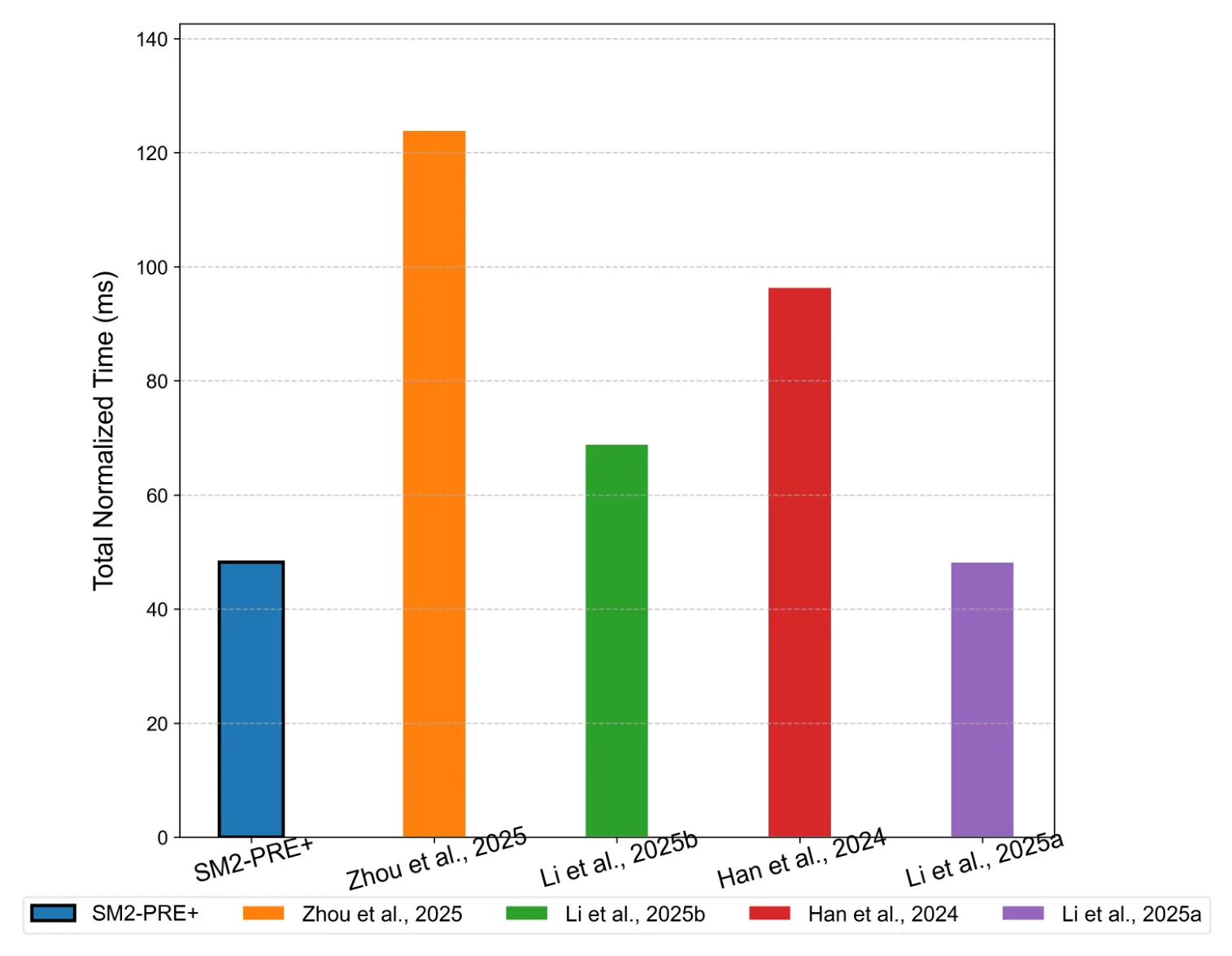
Overall computational overhead comparison of different PRE schemes.The compared competing schemes are from [22,29,41,42].

From Table 5, Figure 3 and Figure 4, it can be observed that the proposed SM2-PRE+ scheme achieves lower computational overhead compared with most existing PRE schemes. In particular, our scheme greatly reduces overhead in the re-encryption phase. The proxy only performs integrity verification and ciphertext transformation, and it does not run costly elliptic-curve scalar-multiplication or pairing operations. Furthermore, the session-key reuse mechanism reduces repeated encryption operations when multiple medical records are shared within one validity window.

Our SM2-PRE+ cryptographic primitives are obtained from real micro-benchmark measurements. In contrast, competing schemes use normalized analytical estimations derived by feeding their reported primitive-operation counts into our measured runtime data, without full end-to-end implementations. Direct porting of some pairing-curve-based reference schemes to SM2 requires non-trivial modifications and would result in unfair benchmarking.

### 6.3. Storage Overhead

Storage overhead is one of the core indicators to measure the practicality of a scheme. In cloud-based healthcare information systems, terminal devices (such as mobile diagnosis and treatment devices, and wearable monitoring devices) generally have limited storage resources, and the high-frequency sharing of medical data will lead to a surge in the storage volume of ciphertexts and keys. Therefore, reducing storage overhead is crucial for the practical deployment of the scheme. This section verifies the advantages of the proposed scheme in resource-constrained scenarios by comparing the storage overhead of the proposed scheme with that of existing typical schemes. This section compares the storage overhead differences between the proposed scheme and related schemes from three dimensions: ciphertext size, re-encrypted ciphertext expansion, and re-encryption key size. The analysis results are shown in Table 6. To unify the measurement standard, the following symbols are defined to represent storage units (with bits in parentheses):

- *G*: SM2 elliptic curve group element (256 bit);
- $G_{1}/G_{2}$: Bilinear pairing group element (512/1024 bit, only used in bilinear pairing schemes, which significantly increases storage overhead);
- $Z_{q}$: Finite field element (256 bit, used for exponentiation or interpolation, increasing key complexity);
- *E*: Symmetric encryption ciphertext (160 bit, medical data encrypted by SM4, simulating actual data of fixed length);
- *H*: Hash value (256 bit, such as SM3, used for integrity verification, a common component of all schemes);
*: Marked for session key reuse (the actual number of storage times is dynamically compressed, and the table shows the element size of a single key);
- $l_{1}+l_{2}$: (160 bit, message length and random string length).

**Figure 5:**
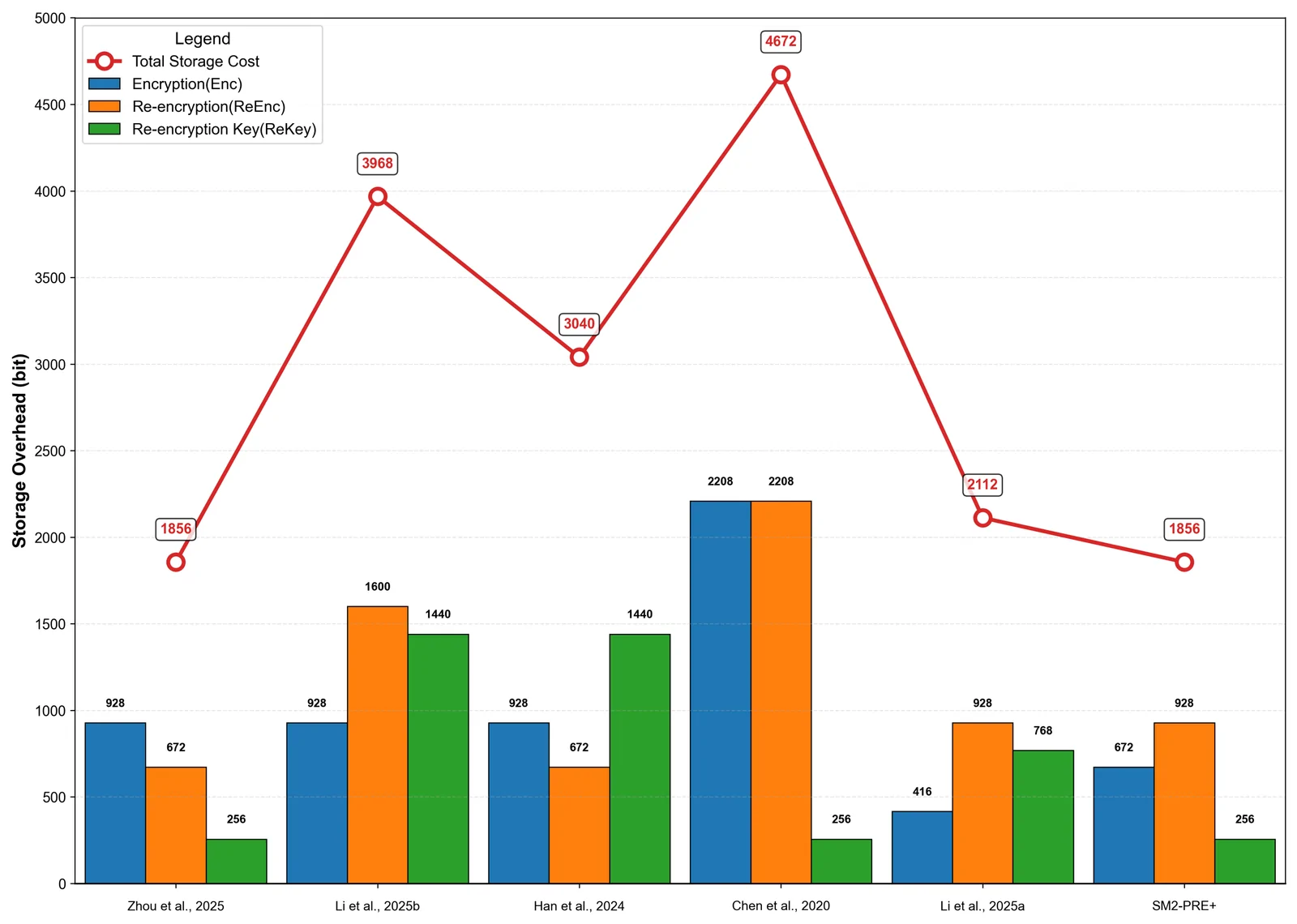
Breakdown of stage-wise and total storage overheads for various schemes.The compared competing schemes are from [21,22,29,41,42].

The total elements output in the three phases. The overall comparison of communication cost is shown in Figure 5.

- In [41] PRE, it is the total cost of communication: $4G+2|l_{1}+l_{2}|+2Z_{q}$.
- In [29] PRE, it is the total cost of communication: $4G+3|l_{1}+l_{2}|+9Z_{q}$.
- In [42] PRE, it is the total cost of communication: $4G+2|l_{1}+l_{2}|+Z_{q}+H$.
- In [21] PRE, it is the total cost of communication: $2G_{1}+2G_{2}+2\left|l_{1}+l_{2}\right|+4Z_{q}+H$.
- In [22] PRE, it is the total cost of communication: $4G+2|l_{1}+l_{2}|+3Z_{q}$.
- In our PRE+ scheme, the overall communication cost is: 4G+E+H.

Under the SM2-256 elliptic-curve standard, the bit-length of core cryptographic objects are specified as follows:

- User public key PK: SM2 elliptic-curve group element, 256 bits.
- User private key sk: Scalar over $Z_{q}^{*}$, 256 bits.
- Re-encryption key $rk_{A\to B}$: SM2 elliptic-curve group element, 256 bits.
- Original ciphertext $C=(C_{0},C_{1},C_{2},C_{3})$: Consists of SM4 ciphertext segments, one SM2 group element, and SM3 integrity hash, total of 792 bits.
- Re-encrypted ciphertext $C^{\prime }=\left(C_{0},C_{1},C_{2},C_{3},D\right)$: Extended from original ciphertext by appending re-encryption-key group element *D*, total of 1048 bits.
- Session-key SK: Output from hash $H_{2}$; actual length is 128 bits matching SM4 symmetric-key requirement. For our storage-overhead formula analysis, a conservative upper-bound value of 256 bits is adopted.

The proposed scheme introduces a long-term Session Key (SK), and its storage overhead model is as follows:

$$S_{SM2-PRE+}\left(N\right)=928N+256$$

In this formula, *N* denotes the total number of shared IoMT-generated medical records. Importantly, this storage model quantifies patient-side local storage overhead, not storage overhead on the cloud-service-provider side. The cloud only stores original ciphertexts; session-key and re-encryption keys are never persisted on cloud servers.

The constant term of 256 bits corresponds to the one-time local storage overhead for the long-lived reusable session-key SK. This session-key is generated once and applies to all medical records within its validity time window, so its storage cost does not grow with the number of medical records *N*. Although the actual output of hash function $H_{2}$ for SM4 session-key is 128 bits, we use a conservative upper-bound of 256 bits for storage evaluation.

The linear-term coefficient of 928 bits represents incremental storage overhead for each additional medical record on the patient trusted terminal. It is the sum of the patient-local stored ciphertext fragment (672 bits) and its corresponding re-encryption key $rk_{A\to B}$ (256 bits), i.e., 672+256=928 bits. Component $C_{2}$ resides on the cloud server and is excluded from patient-side local storage calculation. Each medical record requires its corresponding re-encryption key to be locally kept and indexed with the per-message random scalar *r*, to support potential future re-encryption requests for long-term archived cloud-hosted ciphertexts.

**Figure 6:**
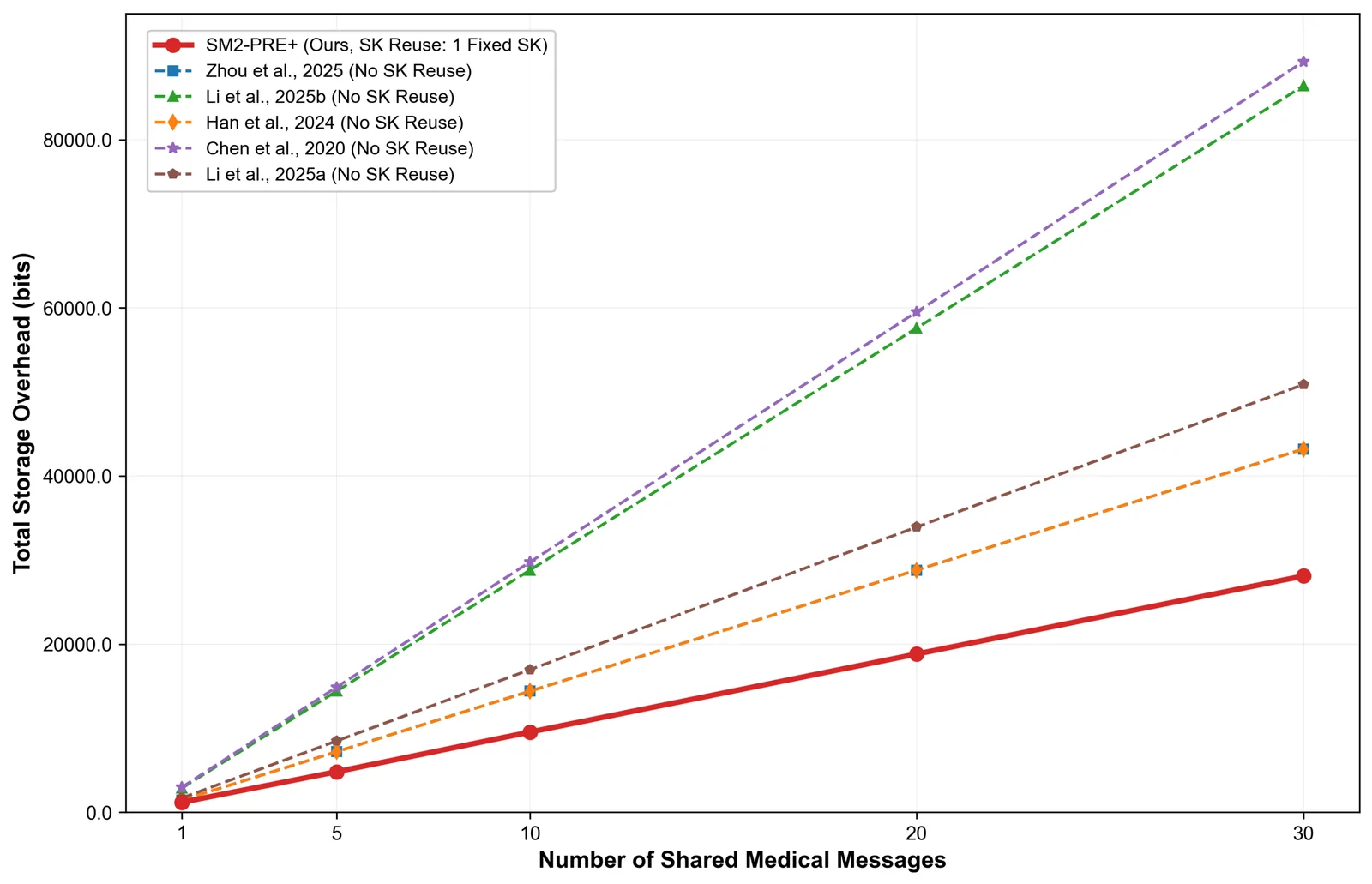
Effect figure of SK reuse.The compared competing schemes are from [21,22,29,41,42].

The effect of session key (SK) reuse is visualized in Figure 6. Benefiting from session-key reuse, our scheme avoids repeatedly storing independent session-key material for every individual medical record. Compared with the “full linear model” $S_{\left[42\right]}\left(N\right)=1440N$ of schemes without SK reuse, the storage slope for a single message in the proposed plan can be greatly reduced, fundamentally avoiding the storage redundancy caused by the “independent key for each message” in traditional schemes.

Detailed bit-size for each cryptographic primitive object can be found in the itemized description above in this subsection.

## 7. Conclusions and Future Work

### 7.1. Conclusions

This paper proposes an SM2-PRE+ secure sharing scheme for IoMT healthcare scenarios. To address core IoMT challenges, including resource-constrained devices and multi-node collaborative security, the scheme leverages pairing-free SM2 elliptic-curve cryptography to lower end-side computational overhead. Built upon the native fine-grained authorization property of PRE+, it achieves message-level non-transferability of access permissions. Furthermore, it tightly binds re-encryption keys to encryption randomness to defend against collusion attacks. Formal security analysis indicates that the proposed scheme satisfies IND-CCA security, collusion-resistance, and non-transferability under the DDH and ECDLP hardness assumptions in the Random Oracle Model. Performance evaluations show that SM2-PRE+ achieves competitive computational efficiency, storage, and communication overhead compared with related existing schemes. This design provides a theoretically feasible cryptographic foundation for resource-constrained IoMT-healthcare scenarios. It demonstrates promising potential for deployment thanks to pairing-free operations and key-reuse functions. Nevertheless, full validation on real medical IoMT hardware and practical system integration belong to future-work directions.

### 7.2. Future Work

Several directions remain for future work. For practical healthcare deployment, we will integrate the scheme with real clinical workflows, perform hardware tests on commercial IoMT medical wearables under ethical approval, and explore third-party compliance evaluation for medical-information systems. From the cryptographic perspective, we aim to develop key-derivation mechanisms for bulk multi-doctor authorization, design instant key revocation independent of re-encryption, and derive security proofs in the standard model to eliminate the Random Oracle Model assumption. For performance evaluation, full end-to-end benchmarking against competing PRE/PRE+ schemes under identical test conditions will complement our current analytical estimations. We will also explore blockchain integration to improve auditability of authorization operations. 

## Figures and Tables

**Table 1:** List of notation symbols.

Symbol	Description
*F*	Prime-order finite field
$Z_{q}^{*}$	Set of all integers in the range [1,q−1]
*G*	Additive cyclic group of prime order *q*
*P*	Generator (base point) of the group *G*
$l_{0}$	Bit-length of the plaintext message domain
$l_{1}$	Bit-length of the random nonce used in encryption
$H_{1}$∼$H_{3}$	Three collision-resistant cryptographic hash functions
$ID_{i}$	Unique identifier of the *i*-th user
$SK_{i}$	Private (secret) key of user *i*
$PK_{i}$	Public key of user *i*
$rk_{A\to B}$	Re-encryption key that transforms a ciphertext from user *A* to user *B*
*C*	Original ciphertext (before proxy transformation) produced by user *i*
$C^{\prime }$	Transformed ciphertext (after re-encryption) intended for the delegatee

**Table 2:** Value assignment of curve parameters.

Curve Parameters	Value Assignment
*n*	FFFFFFFEFFFFFFFFFFFFFFFFFFFFFFFF7203DF6B21C6052B53BBF40939D54123
*p*	FFFFFFFEFFFFFFFFFFFFFFFFFFFFFFFFFFFFFFFF00000000FFFFFFFFFFFFFFFF
$x_{G}$	32c4ae2c1f1981195f9904466a39c9948fe30bbff2660be1715a4589334c74c7
$y_{G}$	bc3736a2f4f6779c59bdcee36b692153d0a9877cc62a474002df32e52139f0a0
*a*	FFFFFFFEFFFFFFFFFFFFFFFFFFFFFFFFFFFFFFFF00000000FFFFFFFFFFFFFFFC
*b*	28E9FA9E9D9F5E344D5A9E4BCF6509A7F39789F515AB8F92DDBCBD414D940E93

**Table 3:** Functional comparison of schemes.

Comparison Dimension	Our Scheme	[41]	[29]	[42]	[21]	[22]
CCA Attack Resistance	*√*	*√*	*√*	*√*	×	*√*
Collusion Attack Resistance	*√*	*√*	×	*√*	×	*√*
Non-transferability	*√*	×	×	×	×	×
Pairing-free	*√*	*√*	*√*	*√*	×	*√*
Key Reusability	*√*	×	×	×	×	×
Fine-grained Authorization	*√*	×	×	×	*√*	×

**Note:***√* indicates that the corresponding property is supported and formally verified; × indicates that the property is not implemented or proven.

**Table 4:** Primitive time consumption table.

Primitive Type	Average Time Consumption (μs)	Number of Rounds
$t_{G}$	6887.38	5000
$t_{A}$	0.47	5000
$t_{H}$	1.34	5000

**Table 5:** Primitive operation counts of each scheme in each phase (ScalarMul, PointAdd, and Hash).

Schemes	SM2-PRE+	[41]	[29]	[42]	[22]
KeyGen	$2t_{G}+2t_{H}$	$2t_{G}$	$2t_{G}+1t_{H}$	$2t_{G}$	$2t_{G}+2t_{H}$
Encrypt	$2t_{G}+2t_{H}$	$3t_{G}+1t_{A}+1t_{H}$	$2t_{G}+2t_{A}+3t_{H}$	$5t_{G}+2t_{H}$	$1t_{G}+4t_{H}$
ReKeyGen	$1t_{G}+1t_{A}$	$1t_{G}+1t_{H}$	$2t_{G}+3t_{H}$	$2t_{G}+1t_{H}$	$1t_{G}+3t_{H}$
ReEncrypt	$1t_{A}+1t_{H}$	$5t_{G}+3t_{A}+3t_{H}$	$1t_{G}+1t_{H}$	$1t_{G}+1t_{H}$	$1t_{G}$
Decrypt	$1t_{G}+2t_{H}$	$3t_{G}+2t_{H}$	$1t_{G}+2t_{H}$	$2t_{G}+2t_{H}$	$1t_{G}+1t_{H}$
ReDecrypt	$1t_{G}+1t_{A}+2t_{H}$	$4t_{G}+3t_{H}$	$2t_{G}+3t_{H}$	$2t_{G}+1t_{H}$	$1t_{G}+5t_{H}$

**Table 6:** Comparison of computational and storage overheads of different schemes. Refer to the itemized textual description in Section 6.3 for concrete bit-length of each cryptographic primitive under SM2-256. Note that our scheme counts patient-side local storage in this table; component $C_{2}$ is stored on cloud and excluded from this table’s calculation.

Schemes	Enc	ReEnc	ReKey	Total Cost
[41]	$2G+|l_{1}+l_{2}|+Z_{q}$	$2G+|l_{1}+l_{2}|$	$Z_{q}$	$4G+2|l_{1}+l_{2}|+2Z_{q}$
[29]	$G+|l_{1}+l_{2}|+2Z_{q}$	$2G+2|l_{1}+l_{2}|+3Z_{q}$	$G+|l_{1}+l_{2}|+4Z_{q}$	$4G+3|l_{1}+l_{2}|+9Z_{q}$
[42]	$2G+|l_{1}+l_{2}|+H$	$2G+|l_{1}+l_{2}|$	$Z_{q}$	$4G+2|l_{1}+l_{2}|+Z_{q}+H$
[21]	$G_{1}+G_{2}+\left|l_{1}+l_{2}\right|+2Z_{q}$	$G_{1}+G_{2}+\left|l_{1}+l_{2}\right|+2Z_{q}$	*H*	$2G_{1}+2G_{2}+2\left|l_{1}+l_{2}\right|+4Z_{q}+H$
[22]	$G+|l_{1}+l_{2}|$	$2G+|l_{1}+l_{2}|+Z_{q}$	$G+2Z_{q}$	$4G+2|l_{1}+l_{2}|+3Z_{q}$
Ours	$G+E^{*}+H$	2G+E+H	*G*	4G+E+H

## Data Availability

Data are contained within this article.
